# Comparative analysis of viral biological characteristics and pathogenicity of representative prevalent avian reovirus strains from genotypes I to V

**DOI:** 10.1080/21505594.2026.2670070

**Published:** 2026-05-08

**Authors:** Fanrun Meng, Ruiqi Li, Dabin Zhang, Haoze Zhou, Chaonan Zhou, Nan Wang, Yang Wang, Fuqiang Guo, Longying Ding, Feng Lang, Liangyu Yang, Ziqiang Cheng

**Affiliations:** a College of Veterinary Medicine, Hebei Agricultural University, Baoding, China; b College of Veterinary Medicine, Shandong Agriculture University, Tai’an, China; c Technology Center, Qingdao Yebio Bioengineering Co., Ltd., Qingdao, China; d College of Veterinary Medicine, Yunnan Agricultural University, Kunming, China

**Keywords:** Avian reovirus, genotype, viral characteristics, B-cell epitope, pathogenicity

## Abstract

The variable pathogenicity of avian reovirus (ARV), driven by genotypic diversity, poses significant control challenges to the global poultry industry. However, biological and pathogenic distinctions among genotypes remain poorly characterized. We performed a comprehensive comparison of 14 ARV strains (genotypes I-V), evaluating viral biological characteristics and pathogenicity indicators. Phylogenetic analysis of these isolates and field strains in China based on the σC gene revealed co-circulation of multiple genotypes (predominantly I and II), with epidemic strains actively evolving and genetically distant from the S1133 vaccine. Structural analysis localized conformational B-cell epitopes of σC protein primarily to the C-terminal globular head and neck, with a highly conserved core antigenic scaffold formed by key residues, while peripheral residues showed genotype-specific variation. Recombination analysis suggests that genotype V (SDAU-G5-DG) likely emerged through recombination with genotype VI. Genotypes I (SDAU-G1-AN4) and IV (SDAU-G4-m4) exhibited enhanced replication and lethality in cell culture and chicken embryos. In vivo, all strains caused growth retardation, lameness, tenosynovitis, and immune organ atrophy, but the tested genotypes I and IV were most virulent. Distinct tissue tropisms were observed: genotypes I and V induced cardiac and hepatic lesions, whereas genotypes I and III caused intestinal damage. Immunologically, genotypes I and IV triggered strong early pro-inflammatory cytokine responses and significantly upregulated MMP13 and Wnt14 at 7 dpi. This points to severe immune activation and a high risk of joint injury. These distinct biological and pathogenic profiles among the tested ARV strains underscore the need for genotype-specific vaccine strategies.

## GRAPHICAL ABSTRACT

**Figure:**
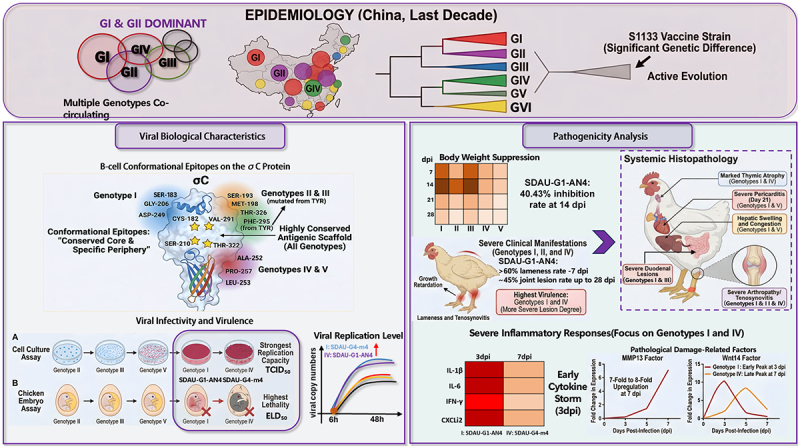

## Introduction

Avian Reovirus (ARV) is characterized as a non-enveloped, double-stranded RNA virus featuring a genome split into ten distinct segments (L1-L3, M1-M3, and S1-S4) [1]. This segmented architecture facilitates significant genetic plasticity [2,3]. Driven by immune pressures from natural infection or vaccination, ARV evolves rapidly via mutation and reassortment. This process leads to the emergence of variant strains with altered antigenicity, posing a persistent challenge to the global poultry industry [4–6]. Within this genome, the S1 gene, which encodes the σC outer capsid protein, serves as the primary determinant of genetic diversity [2]. The σC protein is not only the key receptor-binding protein responsible for viral attachment to host cells but is also the primary antigenic target that induces serotype-specific neutralizing antibodies in the host [7]. Consequently, sequence analysis of the σC gene has become the gold standard for molecular epidemiological studies and genotyping of ARV. Currently, phylogenetic analysis based on the σC gene has classified global circulating ARV strains into at least six major clusters (genotypes I-VI), with historical classic strains, such as the widely used vaccine strain S1133, primarily belonging to genotype I [8,9].

Since the first report of ARV infection in China in 1985, the virus has become significantly endemic within the country. Data from large-scale serological surveys (2010–2024) show a positivity rate of 74.72% in non-vaccinated flocks [10]. Over the past decade, circulating ARV strains in China have diversified into at least six genotypes (I-VI) [11,12]. Concurrently, co-infections with multiple genotypes are commonly observed in clinical samples [13]. From a global perspective, the incidence of ARV has risen significantly in poultry-intensive regions of North America, Europe, and Asia over the last ten years [14]. A major current challenge is the significant genetic divergence between prevalent field strains and traditional vaccine strains like S1133, which poses a potential risk of reduced cross-protection efficacy of existing vaccines. Indeed, surveillance data from China, Brazil, and Europe confirm that non-vaccine genotypes (specifically genotypes II, IV, and V) have become the dominant pathogens responsible for clinical outbreaks [10,15].

ARV infects a wide variety of birds, ranging from chickens and turkeys to ducks and geese [16,17]. The virus spreads through both horizontal (fecal-oral) and vertical (egg-borne) routes [18]. Clinically, infection leads to a spectrum of diseases. The hallmark condition is viral arthritis/tenosynovitis (VA/TS), marked by swollen joints, lameness, and ruptured tendons. Additionally, ARV is linked to malabsorption syndrome, runting-stunting syndrome (RSS), and systemic issues such as myocarditis and hepatitis. These conditions, combined with immunosuppression, inflict heavy economic damage on the global poultry industry [19–21]. It is becoming clear that pathogenicity varies significantly by genotype. Clinical evidence shows that genotype I strains usually drive severe arthritis cases. In contrast, genotypes II and IV are more frequently associated with RSS and systemic organ involvement [22]. Despite this, most research has focused on single strains or sporadic outbreaks. Consequently, a comparative analysis of how different genotypes drive disease is still missing from the literature.

Here, we isolated 14 ARV strains (genotypes I-V) from broiler tendon samples associated with VA/TS. Using these clinically relevant isolates, we systematically compared the biological characteristics and pathogenicity of these genotypes, focusing on genomic evolution, replication capacity, tissue tropism, organ damage, and inflammatory responses. This work not only reveals the complex correlation between ARV genotypic diversity and pathogenic phenotypes through comprehensive data, providing a key theoretical basis for elucidating ARV pathogenesis, but also lays a solid scientific foundation for developing precise, genotype-matched vaccines to effectively address the challenges posed by viral variation in the poultry industry.

## Materials and methods

### Virus isolation

From 2024 to 2025, tendon samples were collected from broilers exhibiting typical clinical signs of ARV infection, such as lameness and hock joint swelling, in major poultry-producing provinces of China (Shandong, Jiangsu, and Gansu). The samples were homogenized under sterile conditions, filtered, and then inoculated onto Leghorn Male Hepatoma (LMH) cells (ATCC, Manassas, VA, USA) for virus isolation. Viral cultures were harvested when a clear cytopathic effect (CPE) was observed. Total RNA was extracted using TRIzol reagent (Invitrogen, Carlsbad, CA, USA) and reverse-transcribed into cDNA. The full-length σC gene was amplified by PCR using primers designed for different ARV genotypes based on sequences from NCBI (primers: F-CAGTCCCTTGTATCGATGT, R-CGTACGGCGCCACACCTTAGGTAT). Positive PCR products were sent to BGI (Beijing Genomics Institute) for Sanger sequencing for viral identification. To obtain pure viral isolates, individual syncytia formed on LMH cells were picked for viral purification. The isolates were confirmed by PCR, and the purity of the viral strains was further ensured by Sanger sequencing.

### Phylogenetic analysis

To investigate the epidemiological characteristics of ARV in China, a total of 130 complete sequences of the ARV σC gene were analyzed in this study. This dataset comprised 116 reference sequences (2016–2025, China, genotypes I-VI) downloaded from the NCBI Pathogen Detection database, along with 14 strains newly isolated in this study (five genotype I isolates, three genotype II isolates, two genotype III isolates, three genotype IV isolates, and one genotype V isolate). Supplementary Table 1 provides comprehensive metadata for all strains, including isolation dates, locations, and genotypes. To illustrate the spatial and source distribution of these isolates, we utilized the ChiPlot platform (https://www.chiplot.online/) to generate geographical maps and Sankey diagrams. For phylogenetic analysis, full-length σC gene sequences (*n* = 130) were aligned via the MUSCLE algorithm in MEGA X (v10.1.5) [23]. The phylogenetic tree was inferred using the Maximum Likelihood (ML) method, with the substitution model selected based on the Bayesian Information Criterion (BIC). Nodal support was assessed through 1,000 bootstrap replicates, and the final tree topology was rendered using iTOL (v6) (https://itol.embl.de/).

### Molecular characterization and conformational epitope analysis of the σC gene

We assessed genetic diversity by computing identity matrices for the σC gene (both nucleotide and amino acid sequences) using ClustalW within MegAlign (DNASTAR Lasergene). For structural analysis, we chose one representative strain from genotypes I through V. We built initial 3D structures of the σC protein with AlphaFold3 and refined them via the Rosetta protocol for energy optimization. Conformational B-cell epitopes were identified using Epitope3D [24,25], and the resulting structures were visualized using PyMOL v1.8.0.3 (http://www.pymol.org/). Furthermore, to comprehensively assess the conservation and topological specificity of σC antigenic epitopes among different genotypes, ten representative strains were selected from each genotype. Multiple sequence alignments were performed on their core B-cell epitope regions, and WebLogo was applied to generate sequence logos, thereby enabling the visualization of residue-level conservation and the identification of key functional sites [26]. For recombination analysis, the σC nucleotide sequences were systematically screened using the RDP4 software package [27], which integrates seven different algorithms. A potential recombination event was considered a reliable signal if it was supported by at least four algorithms with a Bonferroni-corrected *p*-value <0.05. Key events identified by RDP4 were then visually confirmed using SimPlot software to generate similarity plots [28], which allowed for verification of the recombination event and mapping of breakpoint locations by comparing the recombinant sequence with its putative parental strains.

### Development of a SYBR Green Master I mix qRT-PCR assay for ARV genotypes II-V

To enable the specific quantification of viral loads for different ARV genotypes, a SYBR Green I-based quantitative real-time PCR (qRT-PCR) assay for genotypes II-V was developed, building upon a previously established method for genotype I in our laboratory [4]. We utilized Primer 6 to design primers for conserved σC regions across genotypes II, III, IV, and V. We then cloned the PCR products into the pMD-19T vector (Promega, USA) to create recombinant plasmids, which served as positive controls and purified standards. Ten-fold serial dilutions of the purified plasmid standards were prepared, ranging from 1 × 10^8^ to 1 × 10^1^ copies/μL, to generate a standard curve for quantification. In all qRT-PCR assays, nucleic acids extracted from uninfected LMH cells were included as negative controls to rule out contamination. Both specificity and sensitivity were verified according to established protocols [29].

### Viral biological properties

To evaluate the infectivity of all 14 ARV isolates, their 50% tissue culture infectious dose (TCID_50_) and median embryo lethal dose (ELD_50_) were determined. The TCID_50_ was determined in LMH cells using a microtiter assay with CPE as the endpoint. The ELD_50_ was assessed by inoculating 10-day-old specific-pathogen-free (SPF) chicken embryos via the allantoic cavity route. Both titers were calculated using the Reed-Muench method. To further analyze the viral replication kinetics, one representative high-titer strain was selected from each of the five genotypes (SDAU-G1-AN4 (the accession number: PX738110), SDAU-G2-m2 (PX738109), SDAU-G3-m3 (PX738108), SDAU-G4-m4 (PX738107), and SDAU-G5-DG (PX738106)). Monolayers of LMH cells were synchronously infected at a multiplicity of infection (MOI) of 0.1. Cell cultures were collected at 12, 24, 36, 48, 60, and 72 hours post-infection (hpi). To quantify viral loads and generate one-step growth curves for the tested ARV genotypes I-V, we utilized a SYBR Green I-based qRT-PCR system established in our laboratory, designed to specifically target the respective σC gene. To ensure accurate quantification across different samples, the total RNA concentration was standardized prior to reverse transcription. The cDNA concentration used in each qPCR reaction was unified to 1 μg/μL. Therefore, the absolute viral loads in the tissues were normalized and expressed as log10 copies per μg of total RNA. As viral loads were determined using genotype-specific qRT-PCR assays with slightly varying amplification efficiencies, cross-genotype comparisons reflect overall replication trends rather than strictly absolute quantitative differences.

### Pathogenicity test of representative strains of genotypes I-V ARV

To evaluate the pathogenicity of ARV genotypes I-V, 120 1-day-old SPF chicks were randomly divided into six groups (*n* = 20 per group): five virus challenge groups (SDAU-G1-AN4, SDAU-G2-m2, SDAU-G3-m3, SDAU-G4-m4, and SDAU-G5-DG) and a PBS control group. To simulate a high-dose field challenge and maximize the potential differences in pathogenicity among genotypes, all 1-day-old chicks were inoculated via the footpad route with a dose of 10^5^ ELD_50_ in a 0.2 mL volume of virus suspension. As detailed in Viral biological properties, the virus suspension for each strain was diluted based on its predetermined ELD_50_ titer to ensure an identical infectious dose was administered. All experimental chicks were housed in three-dimensional cage systems. To minimize potential cage effects, cages assigned to different treatment groups were arranged in a staggered pattern on the rearing racks. All experimental procedures were conducted in strict accordance with the guidelines established by the Animal Care and Welfare Committee of Shandong Agricultural University. Following challenge, clinical signs were monitored and recorded daily for 28 days. The rates of lameness, joint lesions, and footpad lesions were calculated. Body weights were measured to calculate the rate of body weight gain inhibition, and cloacal swabs were collected to assess viral shedding dynamics. At 7, 14, 21, and 28 dpi, five chicks from each group were randomly selected for sampling. To correlate in vitro replication with in vivo pathogenicity, viral titers at the late exponential phase (48 hpi) were analyzed. We developed a comprehensive virulence score (CVS), calculated as the average of the peak values of four percentage-based clinical metrics: body weight inhibition, lameness, footpad lesions, and joint lesions. Spearman correlation analysis was then performed between the in vitro peak titers and the CVS for the five representative strains. These chicks were humanely euthanized via cervical dislocation, consistent with Section S3.4 of the AVMA Guidelines for the Euthanasia of Animals, and were immediately subjected to necropsy [30]. For nucleic acid extraction, a 1-cm tendon segment was excised from the identical anatomical position of the joint in each chicken. Subsequently, the transcriptional expression levels of key cytokines (IL-1β, TNF-α, IL-6, IFN-γ), a chemokine (CXCLi2), a matrix metalloproteinase (MMP13), and a signaling pathway molecule (Wnt14) were measured using qRT-PCR. The qRT-PCR experiments were performed on a LightCycler 96 system (Roche, Switzerland). The expression data for all cytokines, MMP13, and Wnt14 were normalized to the endogenous reference gene GAPDH [31]. The relative fold changes in expression compared to the control group (Mock) were calculated using the 2^−ΔΔCt^ method. Samples of leg joints, cecal tonsils, liver, heart, duodenum, thymus, spleen, and bursa of Fabricius were also collected. To minimize subjective bias, both the macroscopic lesion scoring and histopathological evaluations were performed independently by two investigators who were blinded to the experimental group assignments. A portion of each tissue sample was immediately stored at −80°C for subsequent quantification of viral loads by RT-qPCR. The remaining tissues were fixed in 10% neutral buffered formalin, embedded in paraffin, sectioned, and stained with hematoxylin and eosin (H&E) for histopathological examination.

### Statistical analysis

All statistical analyses were conducted using GraphPad Prism 8.0 software (GraphPad Software, La Jolla, CA, USA). The unit of analysis for all in vivo experiments was the individual chicken. To analyze multi-timepoint data from independent samples (e.g. viral loads and cytokine expression), a standard two-way analysis of variance (ANOVA) was performed to assess the main effects of genotype and time, as well as their interaction. For longitudinal data where the same animals were monitored continuously over time (e.g. body weight), a mixed-effects model (REML) was applied to account for intra-subject correlation and accommodate missing data resulting from serial euthanasia. Following the ANOVA or mixed-effects model, Tukey’s multiple comparisons test was applied as a post-hoc test. All quantitative data are presented as the mean ± standard deviation (SD). A *p*-value < 0.05 was considered statistically significant. Significance levels in the figures are indicated as follows: *, *p* < 0.05, **, *p* < 0.01, ***, *p* < 0.001, and ****, *p* < 0.0001, ns (not significant).

## Results

### Genotypic classification and evolutionary analysis of ARV isolates

**Figure 1.**
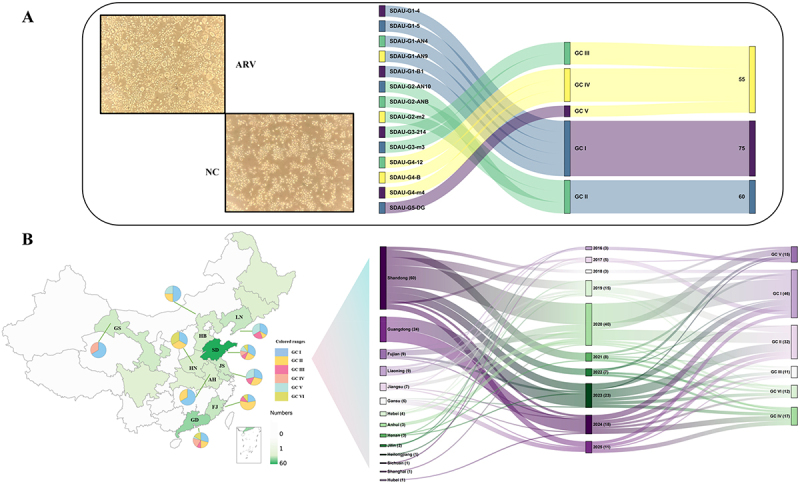
Isolation, characterization, and distribution features of 14 ARV isolates. (A) Left: CPE induced by ARV in LMH cells (100× magnification). Right: genotyping of the 14 ARV isolates. (B) genotypic distribution and evolutionary analysis of ARV in China. The geographic map illustrates the prevalence of ARV isolates (indicated by the color scale) and their genotype compositions (represented by pie charts). A Sankey diagram visualizes the flow of isolates from their province of origin, through the year of isolation, to their assigned genotype. The abbreviations GC I through GC V (genetic clusters I-V) used in the figures correspond to genotypes I to V, respectively, as discussed throughout the manuscript.

Following purification and PCR confirmation, all 14 isolates induced CPE in LMH cells, specifically syncytia formation and cell rounding Figure 1(A). Phylogenetic reconstruction classified these isolates into five distinct genotypes (I-V). Accordingly, the 14 isolates were systematically named with the prefix “SDAU” followed by their respective genotype identifiers.

**Figure 2.**
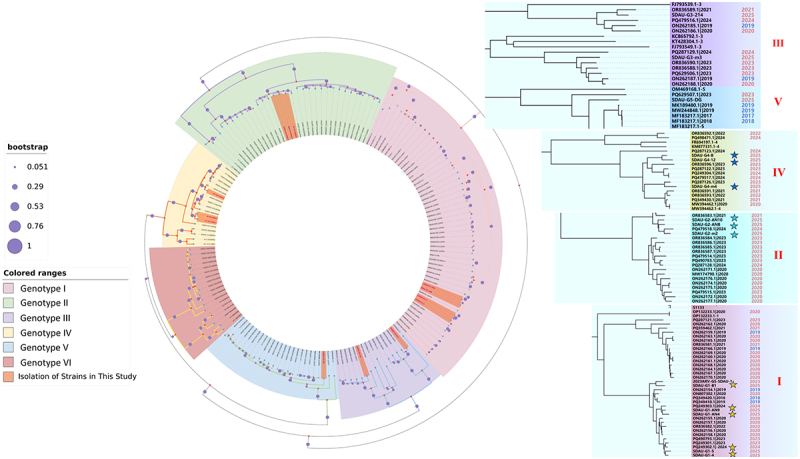
Phylogenetic tree based on the σC gene sequences of ARV isolates (indicated by a star symbol) and Chinese reference strains (2016–2025). The main circular tree on the left is color-coded by genotype (I-VI), and magnified views of key evolutionary branches are shown on the right. The size of the purple circles at the nodes corresponds to the bootstrap support values.

Integrating the sequence characteristics of ARV strains in China over the past decade, this study conducted a sequence dataset-based distribution and evolutionary analysis of the five genotype isolates. The geographical map Figure 1(B) shows widespread ARV distribution in major poultry-producing provinces. Shandong, Guangdong, Fujian, and Jiangsu often harbor four or more co-circulating genotypes, while Gansu shows a simpler pattern, dominated by genotypes I and IV. Over the last decade, genotypes I (*n* = 46) and II (*n* = 32) were the most frequently detected, together comprising 60% of all sequences, followed by genotype IV (*n* = 17), genotype V (*n* = 15), genotype VI (*n* = 12), and genotype III (*n* = 11). The increased number of ARV in 2020 was mainly driven by genotype I and genotype II, indicating their rapid spread during that period. Genotype I, comprising five isolates (SDAU-G1-4, SDAU-G1-5, SDAU-G1-AN4, SDAU-G1-AN9, SDAU-G1-B1), formed a major branch genetically distinct from the classic S1133 strain (Figure 2). These isolates exhibited close affinity with 2023–2024 field strains, suggesting they evolved from recently circulating predominant lineages. Genotype II (SDAU-G2-AN10, SDAU-G2-ANB, SDAU-G2-m2) similarly reflected recent evolution; notably, SDAU-G2-m2 formed a sister branch with a 2024 reference strain, while the others shared ancestry with a 2021 strain. Genotype III isolates (SDAU-G3-214, SDAU-G3-m3) displayed significant divergence, originating from separate subgroups linked to 2021 Shandong and 2024 Guangdong lineages, respectively. Genotype IV (SDAU-G4-12, SDAU-G4-B, SDAU-G4-m4) bifurcated into two subgroups but remained closely associated with isolates from the last two years. Finally, the single genotype V isolate (SDAU-G5-DG) clustered with 2017–2019 strains, representing a lineage that has persisted and continuously evolved in China.

### Viral biological characteristics

#### Homology analysis of the σC gene

**Figure 3.**
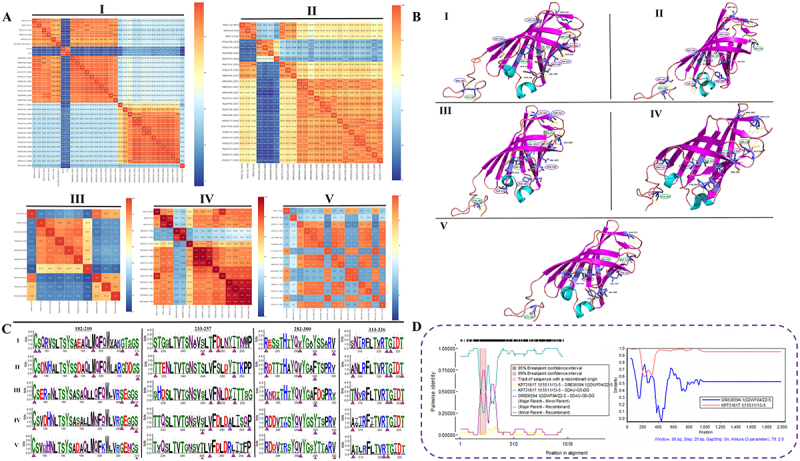
Genetic variation, epitope mapping, and recombination of the ARV σC gene. (A) Identity heatmap of σC nucleotide (lower-left) and amino acid (upper-right) sequences across genotypes I-V. (B) 3D structures and B-cell epitope mapping of representative ARV strains: SDAU-G1-AN4 (genotype I), ON262177.1 (genotype ii), OR836590.1 (genotype iii), MW394462.1 (genotype IV), and PQ287127.1 (genotype V). Secondary structures: α-helices (cyan), β-sheets (magenta), and loops (wheat). Yellow sticks indicate Epitope3D-predicted conformational epitopes on the globular head surface. (C) WebLogo analysis of epitope conservation based on 10 representative strains per genotype. Stack height represents sequence conservation; triangles mark identified epitopes. (D) recombination analysis of strain SDAU-G5-DG. RDP4 (left) and SimPlot (right) identify KP731617.1 and OR836594.1 as major and minor parents, respectively, showing breakpoints and similarity shifts.

Based on sequence analysis, genotype I strains were distinctly partitioned into two major genetic clusters, visualized on the heatmap as high-homology regions (red/orange) separated by a distinct low-identity zone (blue) Figure 3(A). Nucleotide identity between these two clusters dropped markedly to 83.18%-86.85% (see Supplementary Table 2 for identity details). The five genotype I isolates analyzed in this study exhibited high internal nucleotide (95.41%-98.88%) and amino acid (94.5%-98.47%) identities. Although they shared only 75% identity with the classic S1133 strain, their high similarity (95.11%-99.08%) to recent prevalent strains (e.g. PQ249301.1) places them within the currently predominant genotype I lineage. Genotype II isolates displayed high internal conservation (nt: 97.04%-98.27%; aa: 97.25%-98.78%), with identity to other prevalent strains ranging from 83.18% to 98.78%. The two genotype III isolates were significantly divergent, sharing only ~70% identity, and showed broad variation (68.5%-96.94%) against other strains. Genotype IV isolates exhibited internal identities of 91.64%-92.97% and ranged from 78.59% to 97.55% against prevalent strains. Finally, SDAU-G5-DG exhibited the lowest identity (60.55%-93%) with genotype V reference strains, underscoring the extensive genetic diversity of the σC gene.

#### Spatial distribution of B-cell conformational epitopes on the σC protein

Integrated analysis utilizing Epitope3D prediction data and AlphaFold3-generated three-dimensional models reveals that the conformational epitopes of the ARV σC protein are predominantly mapped to the solvent-accessible surface of the C-terminal globular head and the neck region (Supplementary Table 3). This distribution exhibits a distinct spatial pattern characterized by a “conserved core and specific periphery.” Specifically, strains of all five genotypes (I-V) share a highly conserved core antigenic scaffold, comprising CYS-182 located at the neck hinge region, VAL-291 on the lateral face of the β-sheet of the globular head, SER-210 within the apical loop, and THR-322 at the C-terminal base Figure 3(B). The structural stability of these residues suggests they serve as universal epitope anchors across genotypes. However, beyond the conserved scaffold, variations in key residues drive significant genotypic differentiation. As indicated in purple, specific residues of the representative genotype I strain (SDAU-G1-AN4) σC protein are predominantly distributed within surface hypervariable loops. Notably, the highly exposed apical SER-233 constitutes a dominant antigenic site; flanking residues ASP-249 and THR-247 confer unique physicochemical properties by introducing negative charges and steric hindrance, and together with SER-183, MET-198, and GLY-206, form a surface topology unique to this genotype. These loop regions are not disordered but are reinforced by a dense hydrogen bond network (indicated by yellow dashed lines) surrounding ARG-282 and ASP-249. In genotype II, the apical THR-233 replaces SER-233, increasing side-chain volume and steric hindrance; in the basal region, PHE-295 and THR-326 construct a hydrophobic/electrostatic pocket, while SER-193 and MET-198 in the neck region refine the lateral profile. For genotype III, the apical hypervariable region is enriched with positively charged LYS-282 and polar THR-313, creating a distinct electrostatic potential difference compared to genotypes I and II. Flanking ASN-249 replaces ASP-249, eliminating the negative charge and, in coordination with SER-206, remodeling the recognition interface; the basal region retains the PHE-295/THR-326 combination. Genotype IV introduces PRO-257 in the flanking region, where its cyclic side chain restricts backbone degrees of freedom, imparting high conformational rigidity; additionally, GLY-206 and ALA-252 are distributed across different loops, enriching the topology of antigenic epitopes. Genotype V similarly contains PRO-257 in the flanking region, reducing flexibility. In the adjacent domain, LEU-253 introduces hydrophobic bulk, which, combined with surface THR-245, constructs a unique lateral recognition feature.

#### Sequence variation analysis of the identified epitopes

Based on the predicted epitope sites indicated by the triangles in the WebLogo plots and an in-depth analysis of amino acid physicochemical properties, CYS-182/SER-210 (at the start of the 182–210 region), VAL-291 (in the middle of the 282–300 region), and THR-322 (within the 313–326 region) exhibited exceptionally high bit scores (>3.0) and near-absolute conservation across all five genotypes Figure 3(C). Although amino acid residues at key sites exhibit high conservation across genotypes, their potential for recognition as B-cell epitopes displays significant differentiation. Specifically, within the 182–210 region, the genotype I ARV σC protein forms a specific B-cell epitope at the highly conserved residue 183. Concurrently, residue 193, located in the neck hinge region, exhibits sequence polymorphism. While this site is occupied by ALA in other genotypes, it is mutated to SER in genotype II. This mutation within a structurally flexible region may influence local dynamic characteristics. A similar evolutionary trend is observed in the 282–300 region, where the highly conserved residue 282 undergoes a conservative mutation from ARG to LYS in genotype III. Notably, the 233–257 region displays the highest degree of sequence heterogeneity and epitope diversity, characterized by the variation between SER (genotype I) and THR (genotype II) at residue 233, as well as significant variations at residues 245 and 247. Furthermore, the correlation between sequence analysis and structural modeling is strongly corroborated at residue 295. Genotypes I, IV, and V maintain a highly conserved polar TYR at this position, whereas genotypes II and III exhibit a complete mutation to nonpolar PHE. This side-chain modification, accompanied by the loss of a hydroxyl group, directly alters local physicochemical properties, aligning perfectly with structural model predictions regarding the remodeling of the hydrophobic surface in this region. Conversely, conserved residues at critical sites across genotypes have established specific patterns of differentiation, as exemplified by GLY-323 and THR-326 within the 313–326 interval.

#### Recombination events of the σC gene

Recombination analysis of the σC gene sequences from the 14 ARV isolates revealed a clear recombination signal exclusively in the genotype V strain, SDAU-G5-DG; no significant events were detected in the σC genes of the other 13 strains. The σC gene of SDAU-G5-DG was identified as a mosaic sequence consistent with inter-genotypic recombination, with the σC sequence of the USA-derived genotype V strain KP731617.1 serving as the putative major parent and that of the Shandong genotype VI strain OR836594.1 as the minor parent Figure 3(D). Recombination breakpoints were located at 189–202 bp and 239–248 bp (95% CI), consistent with SimPlot analysis which pinpointed them near 197 bp and 242 bp. Therefore, it is hypothesized that recombination within the σC gene of the genotype V strain SDAU-G5-DG has contributed to its genetic diversity.

#### Viral infectivity and virulence

**Figure 4.**
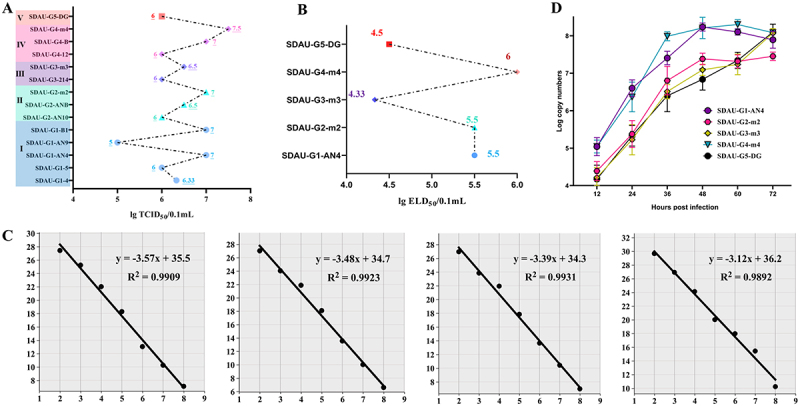
Biological characterization and quantification of ARV isolates. (a) Determination of the TCID_50_ for the 14 ARV isolates in LMH cells. (B) determination of the ELD_50_ for five representative ARV strains. (C) Standard curves for the SYBR Green I qRT-PCR assays developed for ARV genotypes ii, iii, IV, and V (from left to right). The plots show a strong linear correlation between C_t_ values and the logarithm of the template copy number. (D) growth kinetics of the five representative ARV strains in LMH cells. Viral loads at the indicated time points (12 to 72 hpi) were quantified via qRT-PCR. Data are presented as the mean ± standard deviation (SD) of three independent biological replicates (*n* = 3).

To comprehensively evaluate the biological activity of the 14 ARV isolates, we determined their TCID_50_ in LMH cells and ELD_50_. As shown in Figure 4(A), the TCID_50_ titers of the strains ranged from 5.0 to 7.5 log_10_/0.1 mL. The genotype I strain SDAU-G1-AN9 had the lowest titer (5.0 log_10_/0.1 mL), whereas the representative genotype IV strain, SDAU-G4-m4, had the highest titer at 7.5 log_10_/0.1 mL, demonstrating a very high in vitro infectivity. To further assess their in vivo virulence, we determined the ELD_50_ of five representative strains Figure 4(B). The results again indicated that the representative genotype IV strain, SDAU-G4-m4, was the most lethal to chicken embryos, with an ELD_50_ titer as high as 6.0 log_10_/0.1 mL. In contrast, the genotype V recombinant strain SDAU-G5-DG and the representative genotype III strain SDAU-G3-m3 were relatively less lethal to chicken embryos, with ELD_50_ titers of 4.5 and 4.33 log_10_/0.1 mL, respectively.

#### Viral replication level

To enable the precise quantification of ARV genotypes I-V, we successfully developed SYBR Green Master I qRT-PCR assays targeting the σC gene of genotypes II, III, IV, and V (See Supplementary Table S4 for detailed primer information). Over a wide detection range, a strong linear relationship was observed between the C_t_ values and the logarithm of the initial template copy number. The linear regression equations and correlation coefficients (R^2^) for the standard curves of each genotype were as follows Figure 4(C): genotype II (Y = −3.57x + 35.5, R^2^ = 0.9909), genotype III (Y = −3.48x + 34.7, R^2^ = 0.9923), genotype IV (Y = −3.39x + 34.3, R^2^ = 0.9931), and genotype V (Y = −3.12x + 36.2, R^2^ = 0.9892). Detailed validation results regarding the assay sensitivity (detection threshold of 1 × 10^1^ copies/µL), specificity, and melting curve analyses are provided in Supplementary Figure S1. One-step growth curve analysis was performed on five representative strains (SDAU-G1-AN4, SDAU-G2-m2, SDAU-G3-m3, SDAU-G4-m4, and SDAU-G5-DG) in LMH cells to characterize their replication kinetics. All five representative strains replicated efficiently, with viral loads rising significantly starting from 12 hpi Figure 4(D). Notably, SDAU-G1-AN4 and SDAU-G4-m4 exhibited the most robust replication kinetics, rapidly reaching peak titers of approximately 10^8.2^ copies by 48 hpi. While SDAU-G3-m3 and SDAU-G5-DG displayed a steadier increase to reach comparable high titers by 72 hpi, SDAU-G2-m2 showed the lowest overall replication efficiency, plateauing at approximately 10^7.5^ copies.

### Pathogenicity indicators

#### Clinical manifestations

**Figure 5.**
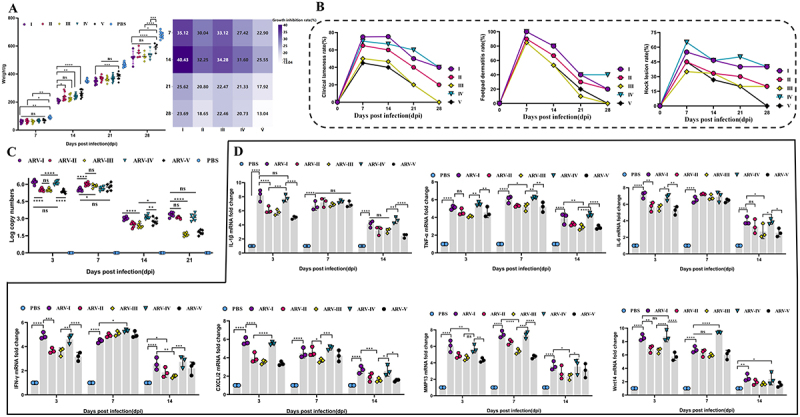
Pathogenicity of ARV isolates representing genotypes I-V in chickens. (a) Body weight changes (left) and growth inhibition rates (heatmap, right) of chickens within 28 dpi. The body weight change data reflect measurements from 10 randomly selected surviving chickens at each time point, with data at 28 dpi obtained from the remaining 5 chickens. (b) dynamic monitoring of clinical signs, including rates of clinical lameness, footpad lesions, and hock joint lesions. Sampling was performed on all surviving chickens at each time point. (C) Quantification of viral shedding in cloacal swabs at 3, 7, 14, and 21 dpi. (d) Relative mRNA expression levels of key immune- and pathology-related factors in hock joint tissue at 3, 7, and 14 dpi. Statistical significance was determined using a two-way ANOVA followed by Tukey’s multiple comparisons test. Statistical significance is indicated as: *p < 0.05, **p < 0.01, ***p < 0.001, and ****p < 0.0001, ns (not significant). The label “ARV-I” (and similarly for II-V) denotes the specific representative strain belonging to genotype I (and respective genotypes) evaluated in this study.

To evaluate the influence of representative ARV genotypes I-V on chick development, clinical signs and growth performance were monitored over 28 days. Compared to the PBS control, all infected groups showed significantly reduced body weights, highlighting the growth-inhibitory effect of ARV. The highest pathogenicity was observed in the genotype I strain (SDAU-G1-AN4), which caused a maximum weight loss of 40.43% at 14 days post-infection (dpi), while the genotype III strain (SDAU-G3-m3) led to a reduction of 34.28% Figure 5(A). Clinical signs appeared at 7 dpi, peaked between days 7 and 14, and subsequently subsided. Strains of genotypes I, II, and IV caused the most severe symptoms, with lameness rates exceeding 60% at 7 dpi. Footpad lesions affected approximately 40% of the animals in these groups up to 21 dpi, indicating slow healing Figure 5(B). Furthermore, hock joint lesions in the tested genotypes IV and I groups remained at a high level (~45%) until 28 dpi, approximately 20 percentage points higher than those in the other groups. To assess the correlation between in vitro replication and in vivo pathogenicity, we analyzed the peak viral titers (48 hpi) and the CVS of five representative strains using Spearman’s rank correlation. The 48 hpi viral titers for the genotypes I-V ARV strains were 8.23, 7.38, 7.09, 8.21, and 6.84 log_10_ copies, corresponding to CVS values of 67.61%, 58.06%, 54.82%, 66.65%, and 50.14%, respectively. Although, the analysis demonstrated a perfect positive correlation between these two parameters (Spearman *r* = 1.000, *p* = 0.017), this finding should be considered an exploratory observation given the limited panel of five strains tested.

#### Cloacal viral shedding

Viral shedding analysis showed that genotype I (SDAU-G1-AN4) and genotype IV (SDAU-G4-m4) strains had maximum viral copy numbers (approx. 6.0 log_10_ copies) at 3 dpi Figure 5(C). Viral shedding in these two groups was significantly higher than in the other groups (****p < 0.0001), with no significant difference observed between them (ns). On the contrary, the peak of viral shedding in genotypes II (SDAU-G2-m2), III (SDAU-G3-m3), and V groups occurred at 7 dpi. In the subsequent week, shedding began to decrease in all groups, albeit with a slight rebound observed in the genotypes I and II strains.

#### Levels of inflammation and tissue injury-related factors

To assess the host immune response and pathogenic potential of each virus strain, key immunological and pathological parameters were measured. At 3 dpi, chicks infected with genotypes I (SDAU-G1-AN4) and IV (SDAU-G4-m4) strains had the highest levels of pro-inflammatory factors IL-1β (>7.5-fold), IL-6 (>6.5-fold), IFN-γ (>4.5-fold), as well as the neutrophil chemokine CXCLi2 (>5.5-fold) at this timepoint. The peak expression levels in the other groups occurred by 7 dpi Figure 5(D). Genotypes I and IV induced significant peaks that were higher than those of the other genotypes. This indicates a stronger early inflammatory response. We further analyzed the expression of specific molecules in joint tissue during the infection. Notably, the expression of MMP13, an enzyme involved in tissue degradation, peaked at 7 dpi in the genotypes I and IV groups. These groups exhibited a 7- to 8-fold increase, which was significantly higher than that of the other groups (***p < 0.001, ****p < 0.0001). In order to monitor upstream regulation, we measured Wnt14. Wnt14 is a signaling molecule that plays a key role in joint homeostasis. The tested genotype I caused Wnt14 to be upregulated significantly early on (3 dpi), while genotype IV caused an approximate 10-fold increase at 7 dpi when MMP13 was maximally expressed. The genotypes II (SDAU-G2-m2), III (SDAU-G3-m3), and V (SDAU-G5-DG) resulted in significantly lower levels.

#### Tissue tropism and damage

**Figure 6.**
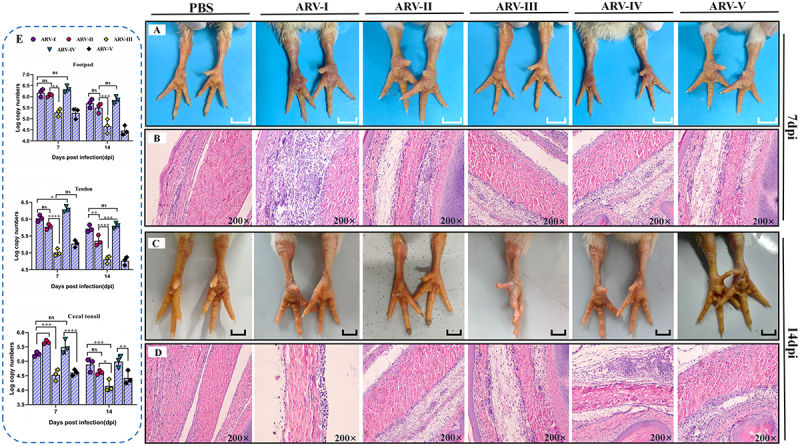
Pathological lesions and viral replication of ARV isolates representing genotypes I-V in joints. SPF chicks were infected with ARV-I (SDAU-G1-AN4), ARV-II (SDAU-G2-m2), ARV-III (SDAU-G3-m3), ARV-IV (SDAU-G4-m4), or ARV-V (SDAU-G5-DG), with a PBS-injected group serving as the control. (A, C) gross lesions in the footpads and hock joints at 7 dpi and 14 dpi, respectively. (B,D) histopathological changes in tendon tissues at 7 dpi and 14 dpi, respectively, visualized by H&E staining. (E) Viral loads in the footpad, tendon, and cecal tonsils at 7 and 14 dpi, as determined by qPCR. Data are presented as the mean ± standard deviation (sd) of three independent biological replicates (*n* = 3). Statistical significance was determined using a two-way ANOVA followed by Tukey’s multiple comparisons test. Statistical significance is indicated as: *p < 0.05, **p < 0.01, ***p < 0.001, and ****p < 0.0001, ns (not significant).

Animal experiments were conducted to investigate the relationship between virulence, tissue tropism, and replication kinetics among different ARV genotypes. Necropsy at 7 and 14 dpi revealed that genotypes I (SDAU-G1-AN4) and IV (SDAU-G4-m4) strains caused the most severe joint pathology, with marked swelling of the footpads and hocks Figure 6(A,C). Histopathological analysis confirmed tenosynovitis in all infected groups, characterized by peritendinous infiltration of lymphocytes and heterophils, edema, disorganized connective tissue and tendon fibers, and scattered inflammation (Figure 6(B)). By 14 dpi, infections with the tested genotypes I and IV had progressed to more severe lymphocytic infiltration and tendon rupture (Figure 6(D)). Consistent with these findings, viral loads in footpads and tendons at 7 dpi were highest in chicks infected with genotypes I, II, and IV (≈5.6–6.5 log_10_ copies) (Figure 6(E)), significantly exceeding those of genotypes III and V (**p < 0.01). Although viral loads declined by 14 dpi, they remained highest in the genotypes I and IV groups (≈5.5–6.0 log_10_ copies). In the cecal tonsils, a common site for clinical sampling, genotypes II and IV replicated most efficiently (≈5.5 log_10_ copies).

**Figure 7.**
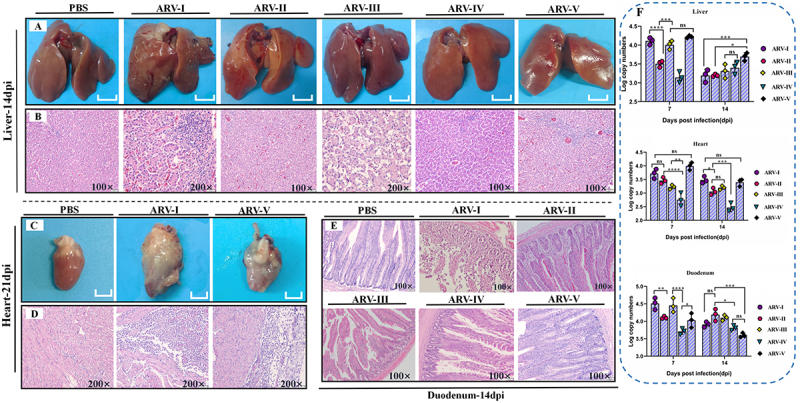
Pathological lesions and viral replication of ARV isolates representing genotypes I-V in visceral organs. (A, B) gross lesions and corresponding histopathological changes in the liver at 14 dpi. (C, D) gross lesions and histopathological changes in the heart of the PBS, ARV-I, and ARV-V groups at 21 dpi. (E) histopathological changes in the duodenum at 14 dpi. (F) viral loads in the liver, heart, and duodenum at 7 and 14 dpi, as determined by qPCR. Statistical significance is indicated as: *p < 0.05, **p < 0.01, ***p < 0.001, and ****p < 0.0001, ns (not significant).

**Figure 8.**
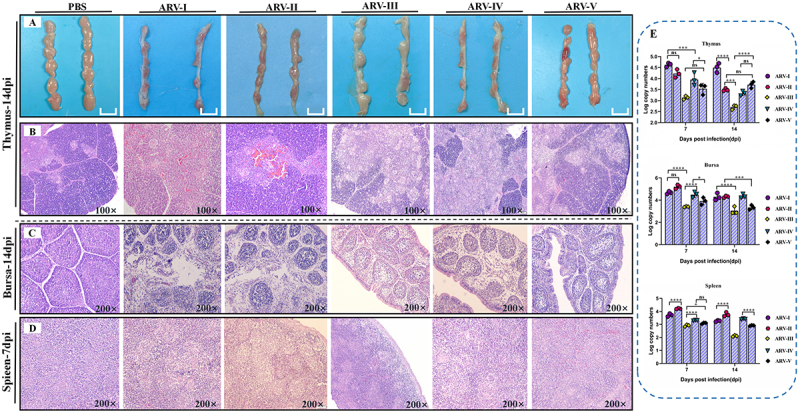
Pathological lesions and viral replication of ARV isolates representing genotypes I-V in immune organs. (A, B) Gross lesions and corresponding histopathological changes in the thymus at 14 dpi. (C) histopathological changes in the bursa of Fabricius at 14 dpi. (D) histopathological changes in the spleen at 7 dpi. (E) viral loads in the thymus, bursa of Fabricius, and spleen at 7 and 14 dpi, as determined by qPCR. Statistical significance is indicated as: * *p* < 0.05, ***p < 0.001, and ****p < 0.0001, ns (not significant).

Systemic pathology also varied by genotype. At 14 dpi, livers from the genotypes I and V groups showed swelling and congestion (Figure 7(A)), with histological evidence of disordered hepatic cords, sinusoidal congestion, and perivascular inflammation (Figure 7(B)). In contrast, genotype III induced more pronounced hepatocyte degeneration. By 21 dpi, genotypes I and V had induced severe pericarditis (Figure 7(C)), which manifested as interstitial myocarditis, lymphocytic infiltration, epicardial thickening, and mild fibrosis (Figure 7(D)). Furthermore, genotype I inflicted severe duodenal damage, including complete shedding of the villous epithelium, loss of gland cells, and a reduced number of intestinal glands (Figure 7(E)), whereas genotype III primarily caused villous fracture with minimal shedding. Pathological changes were also evident in immune organs. Genotypes I and IV caused marked thymic atrophy (Figure 8(A)), characterized by cortical thinning and lymphocyte depletion (Figure 8(B)), while genotype II infection resulted in rare medullary congestion. All challenged groups exhibited bursal atrophy with follicular shrinkage and lymphocyte loss (Figure 8(C)). Splenic lesions were generally mild across groups, with the exception of genotype II, which caused indistinct red and white pulp boundaries (Figure 8(D)). Overall, peak viral loads in non-joint tissues ranged from 2.5 to 5.5 log_10_ copies (Figure (7F,8E)), levels lower than those in tendons and footpads, and generally paralleled the severity of pathological lesions.

## Discussion

The rapid genetic evolution and constant emergence of novel pathogenic variants make ARV a persistent challenge for the global poultry industry, complicating current control efforts [32,33]. In this study, we isolated and identified 14 prevalent strains across genotypes I-V in China. We then compared their viral biological characteristics and pathogenicity. This work provides the essential evidence base needed to refine prevention and control measures against ARV. Genotype VI was excluded from this study due to the scarcity of clinical cases. Recent epidemiological surveys indicate that genotype VI remains rare in China compared to the predominant circulating genotypes [11], limiting the availability of field strains for comparative experimental infection.

To ascertain the evolutionary placement of the 14 ARV isolates in the context of currently circulating strains, we first performed a phylogenetic analysis based solely on the σC gene. The result revealed that the 14 new strains isolated in this study were interspersed among recent Chinese strains, occupying terminal branches of the tree. Current epidemic strains are distantly related to the classic vaccine strain S1133 but are closely related to strains from 2021–2024, suggesting continuous evolution of this specific segment, consistent with previous reports [34]. Furthermore, significant genetic divergence within genotypes (e.g. I and IV) suggests the existence of multiple independent transmission chains rather than directional evolution from a single origin.

The σC protein is the key protein responsible for host cell attachment and the induction of neutralizing antibodies; therefore, variation in its antigenic structure is a core mechanism of viral immune evasion [35]. Previous studies have primarily identified linear epitopes (e.g. amino acids 45–58 of strain S1133) [36]. However, it is well established that over 90% of natural B-cell epitopes are discontinuous conformational epitopes, which are crucial for eliciting potent neutralizing antibodies [37]. Through an integrated analysis of Epitope3D predictions and AlphaFold3 structural modeling, this study identified a “conserved core and specific periphery” pattern in the epitope distribution of the σC protein. A conserved antigenic scaffold, comprising CYS-182, VAL-291, SER-210, and THR-322, exhibits exceptionally high bit scores and near-absolute conservation across all five genotypes. The scaffold located at the neck hinge and key sites of the globular head resembles conserved regions like the influenza virus HA protein stem [38]. While this conserved core represents a candidate target for cross-genotype vaccine design, such a universal approach remains speculative at this stage. On the other hand, the peripheral regions of the σC protein exhibit significant divergence. For instance, the substitution of THR-233 in genotype II (vs. SER-233 in genotype I) is predicted to increase steric hindrance, while the TYR-to-PHE mutation at position 295 in genotypes II and III may alter local hydrophobicity, potentially influencing recognition by neutralizing antibodies [39,40]. However, it is essential to emphasize that these predicted epitopes and their functional relevance require rigorous experimental validation, such as monoclonal antibody mapping or cross-neutralization assays using sera against different genotypes. In conclusion, these structural insights offer theoretical concepts that may serve as a reference for future antigen design, such as developing chimeric σC proteins that combine a conserved scaffold with genotype-specific loops, with the theoretical aim of enhancing cross-protective efficacy.

Point mutations and recombination are generally recognized as primary drivers of viral evolution. Similar to other RNA viruses such as SARS-CoV-2 [41,42], these mechanisms are critical for the genetic diversification and emergence of novel variants in ARV [11,43]. In this study, no clear recombination signals were detected in the strains belonging to genotypes I-IV. In contrast, the σC gene of the genotype V strain, SDAU-G5-DG, exhibited distinct mosaic characteristics, suggesting the occurrence of intragenic recombination. These findings align with previous studies, confirming that recombination acts as a significant mechanism contributing to ARV genetic diversity [34]. However, our conclusions regarding the genotype V origin are strictly limited to the σC gene and should not be overinterpreted without broader sampling. Additionally, excluding genotype VI leaves a gap in our comparative framework. Future whole-genome sequencing is required to determine whether similar recombination events affect other genomic segments.

The in vitro viral replication levels and in vivo viral shedding dynamics observed in our study are consistent with the findings of previous ARV transcriptomic analyses [6,33]. More significantly, spearman correlation analysis revealed a significant positive correlation between the peak viral titers of the five representative strains and their corresponding clinical severity metrics. Specifically, the tested genotypes I and IV strains exhibited robust replication capacities, which correspondingly manifested as more severe clinical disease and prolonged viral shedding patterns, aligning well with earlier reports [44,45]. From a clinical perspective, this exploratory observation suggests that in vitro replication capacity has the potential to serve as a rapid screening marker for virulence. However, given the limited number of strains tested in this study, validation with a much larger strain panel is required before it can be established as a general predictive tool for early warning and targeted prevention during outbreaks.

ARV primarily causes viral arthritis in broilers. As all 14 isolates were exclusively derived from VA/TS-affected tendons, our findings primarily reflect these joint-tropic strains rather than broader enteric/respiratory lineages. Although the footpad challenge in 1-day-old SPF chicks is a widely established laboratory model for evaluating ARV pathogenicity, it may not fully replicate natural infection routes or true field virulence. Within this context, genotypes I (SDAU-G1-AN4) and IV (SDAU-G4-m4) exhibited the highest clinical pathogenicity. Their severe tenosynovitis phenotypes were closely linked to rapid viral replication in joints and footpads. These strains induced an early and intense inflammatory storm, marked by a 4.5–7.5-fold increase in IL-1β, IL-6, and CXCLi2 expression by 3dpi, thereby worsening edema and tissue damage [46,47]. As key pro-inflammatory cytokines, IL-1β and IL-6 are important pro-inflammatory factors that increase local vascular permeability and leukocyte recruitment and induce fever [48]. Furthermore, CXCLi2 enhances the recruitment of heterophils (avian neutrophils) to the site of infection, contributing to tissue edema and destruction [49]. Earlier investigations have shown that abnormal upregulation of MMP13 is strongly associated with tendon degeneration, synovial hyperplasia, and articular cartilage damage [50]. It is important to emphasize that MMP13 is a target gene of the Wnt signaling pathway. The Wnt pathway can directly or indirectly induce transcription of MMP13 gene via its core transcription factors (β-catenin/TCF/LEF complex) which ultimately enhances the synthesis and secretion of MMP13 protein [51]. Our findings are in accordance with this conclusion and indicate that strains that are highly pathogenic almost ten-fold increased the expression of Wnt14. At the seventh day post-infection, it was revealed that MMP13 expression was elevated by 7 to 8-fold as pathology peaked. This indicates a strong association between the upregulation of the Wnt14-MMP13 signaling pathway and the severity of tissue damage. Since this peak occurred concurrently with high levels of viral load and release of cytokines, inflammation may enhance MMP13 production [52]. As such, Wnt14 and MMP13 May be potential biomarkers for disease severity. Moreover, our data suggest that targeting the virus and the inflammatory response may be twofold beneficial therapy.

The study of tissue tropism highlighted the aggressiveness of the tested genotypes I and IV. Genotype I was not restricted to the joints, but also severely affected the duodenum, including complete loss of the epithelial villi. In comparison, while genotype IV produced milder symptoms, infected chickens exhibited pronounced atrophy of bursa of Fabricius and thymus tissues indicating a greater immunosuppressive capacity [53,54]. Furthermore, genotypes I and V showed tropism for the heart and liver. Liver inflammation was visible at 14 dpi, and we were able to detect the virus in myocardium as late as 21 dpi. The cardiac lesions observed only after viremia clearance indicates that ARV is utilizing low surveillance sites for establishment of persistent infection similar to MRV latency [55,56]. Moreover, a consistent trend was observed, wherein higher viral loads were generally accompanied by more severe histopathological damage. The results show viral loads of the tested genotypes I and IV were maintained at a high level, in primary target tissues such as the footpad and tendon. This was several log_10_ higher than that in other tissues. This shows their tissue tropism and efficiency.

Our study has several limitations. In vivo pathogenicity was evaluated using only one representative strain per genotype. Given the virulence heterogeneity within genotypes, the high pathogenicity observed for genotypes I and IV strains cannot be generalized to their entire genotypes without evaluating additional isolates. Finally, without an a priori statistical power calculation, our initial sample sizes were based on standard protocols and animal welfare guidelines. The serial euthanasia required for tissue collection progressively reduced the remaining subjects, potentially limiting the statistical robustness of late-stage observations.

In conclusion, a systematic comparison of different ARV genotypes was conducted at the levels of viral biology and pathogenicity, revealing distinct variations among the strains. Notably, the ARV σC protein exhibits an antigenic pattern featuring a conserved structural core (e.g. CYS-182) and divergent surface loops containing genotype-specific epitopes. Furthermore, based on the evaluated strain cohort in this study, the representative isolates of genotypes I and IV were identified as highly pathogenic strains. Their pathogenic mechanism is closely associated with efficient viral replication, an early and excessive local inflammatory response, and subsequent tissue degradation. These findings not only deepen our understanding of ARV pathogenesis but also provide a crucial theoretical basis and experimental support for the precise diagnosis, virulence assessment, and development of genotype-matched vaccines for this disease.

## Supplementary Material

Supplementary Materials.docx

Supplementary Figure 1B.tif

Supplementary Figure 1A.tif

Supplementary Table.xlsx

## Data statement

The data that support the findings of this study along with the supplementary tables are openly available in Figshare at http://doi.org/10.6084/m9.figshare.30995371, reference number [57].
