# Supplementary material for: Comparative analysis of viral biological characteristics and pathogenicity of representative prevalent avian reovirus strains from genotypes I to V
Source: Virulence. 2026 May 8;17(1):2670070. doi: 10.1080/21505594.2026.2670070 (PMC13166242; doi:10.1080/21505594.2026.2670070)
Supplement: Supplementary Materials.docx [file KVIR_A_2670070_SM0672.docx]

**Supplementary Materials**

**Supplementary Figure 1. II-V ARV Validation of the Genotype-Specific qRT-PCR Assays.**

To accurately quantify viral loads, we developed and rigorously validated SYBR Green I-based qRT-PCR assays specifically targeting ARV genotypes II, III, IV, and V. To evaluate the analytical performance, standard curves were generated using 10-fold serial dilutions of the standard plasmids (A) Based on the slopes of these respective standard curves, the calculated amplification efficiencies were highly optimal, reaching 90.6%, 93.8%, 97.2%, and 99.2% for genotypes II to V, respectively. In terms of assay sensitivity, these serial dilutions demonstrated a highly reliable detection threshold, establishing a strict limit of detection (LOD) at 1×10^1^ copies/μL for all four genotypes; crucially, alongside this high sensitivity, melting curve analysis revealed a single, sharp specific peak for each respective assay, confirming the complete absence of primer dimers or non-specific amplification products even at the lowest detection limits. (B) To rigorously confirm analytical specificity, each genotype-specific assay was tested against its corresponding positive target plasmid as well as a mixed sample containing plasmids of all other non-target genotypes. The results showed robust and exclusive amplification only for the target genotype, with absolutely no cross-reactivity observed from the mixed non-target plasmids, thereby ensuring highly accurate viral quantification.

**Supplementary Table 1:** Source Information for the 130 ARV Isolates.

**Supplementary Table 2:** The pairwise identity (nt/aa %) matrix of different ARV genotypes.

**Supplementary Table 3:** B-cell conformational epitope prediction results.

**Supplementary Table 4:** Primers for SYBR Green I-based qRT-PCR targeting ARV genotypes II-V.
