# Supplementary figures and images for: Comparative analysis of viral biological characteristics and pathogenicity of representative prevalent avian reovirus strains from genotypes I to V
Source: Virulence. 2026 May 8;17(1):2670070. doi: 10.1080/21505594.2026.2670070 (PMC13166242; doi:10.1080/21505594.2026.2670070)

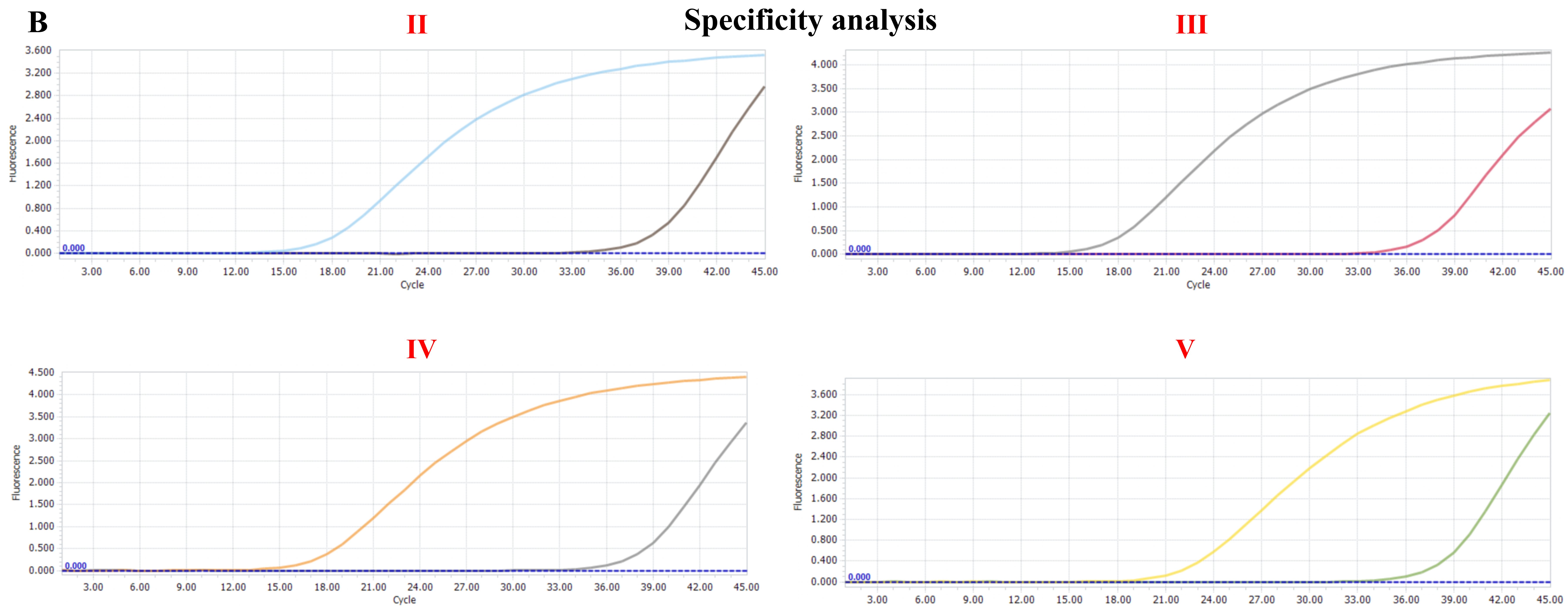

Supplement: Supplementary Figure 1B.tif [file KVIR_A_2670070_SM0671.tif]

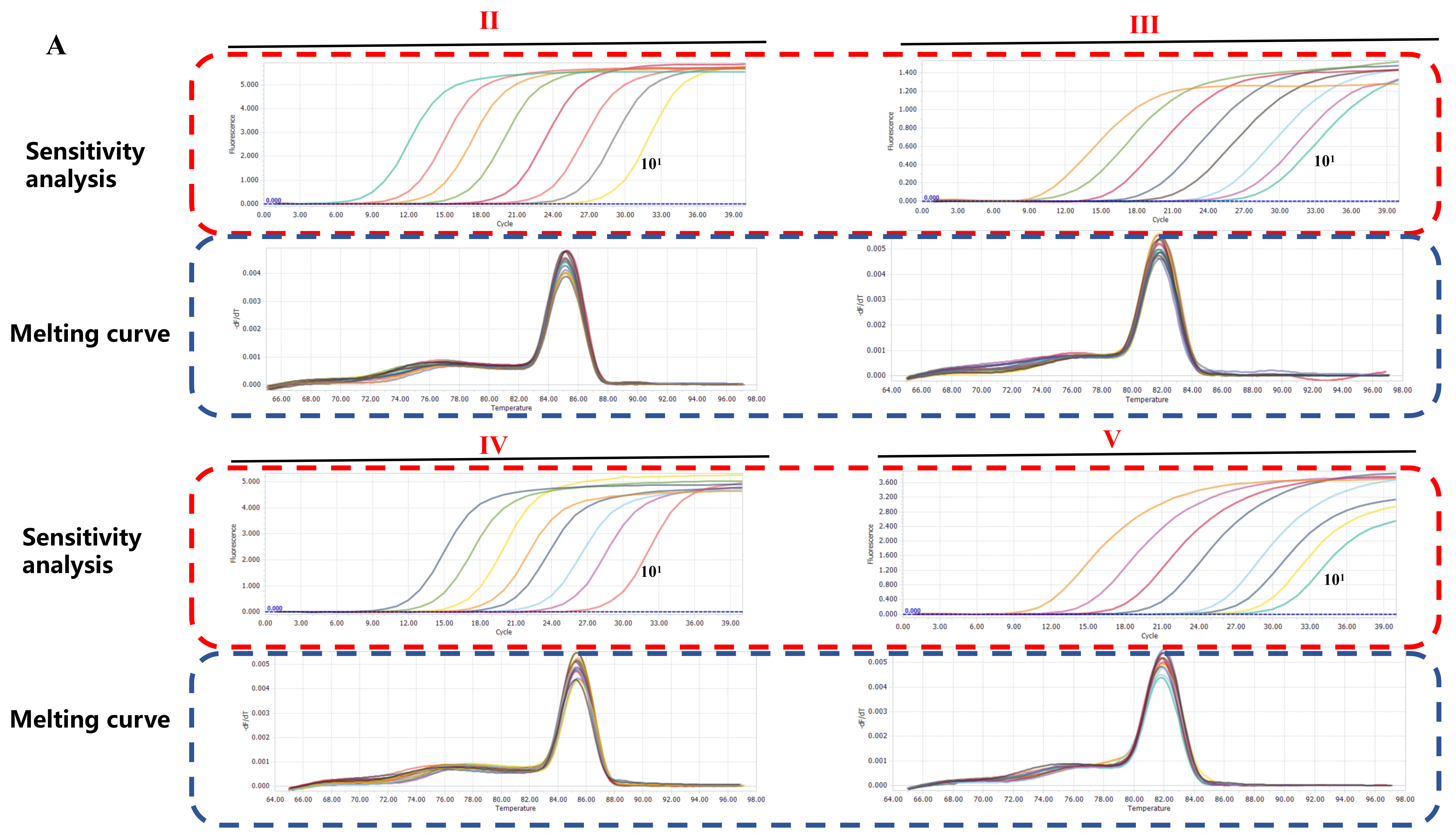

Supplement: Supplementary Figure 1A.tif [file KVIR_A_2670070_SM0670.tif]
